# Exercise-Induced Muscle Damage after a High-Intensity Interval Exercise Session: Systematic Review

**DOI:** 10.3390/ijerph20227082

**Published:** 2023-11-20

**Authors:** Carine D. F. C. Leite, Paulo V. C. Zovico, Roberta L. Rica, Bruna M. Barros, Alexandre F. Machado, Alexandre L. Evangelista, Richard D. Leite, Valerio G. Barauna, Adriano F. Maia, Danilo S. Bocalini

**Affiliations:** 1 Experimental Physiology and Biochemistry Laboratory, Physical Education and Sport Center, Federal University of Espírito Santo, Vitória 29075810, ES, Brazil; 2 Department of Physical Education, Estacio de Sá University, Vitoria 29090640, ES, Brazil; 3 Nove de Julho University, São Paulo 01504000, SP, Brazil; 4 Departament of Physical Education, Ítalo Brasileiro College, São Paulo 04080000, SP, Brazil; 5 Exercise Physiology Laboratory, Physical Education and Sport Center, Federal University of Espírito Santo, Vitória 29075810, ES, Brazil; richard.leite@ufes.br; 6 Department of Physiological Sciences, Federal University of Espírito Santo, Vitoria 29043900, ES, Brazil; valerio.barauna@ufes.br; 7 Laboratory of Nutrition and Metabolism, Physical Education and Sport Center, Federal University of Espírito Santo, Vitória 29043900, ES, Brazil; adriano.maia@ufes.br

**Keywords:** exercise, high-intensity interval training, physical performance, muscle damage

## Abstract

High-intensity interval training (HIIT) is considered an effective method to improve fitness and health indicators, but its high-intensity exercises and the mechanical and metabolic stress generated during the session can lead to the occurrence of exercise-induced muscle damage. Therefore, this study aimed to describe, by means of a systematic review, the effects of a single HIIT session on exercise-induced muscle damage. A total of 43 studies were found in the Medline/PubMed Science Direct/Embase/Scielo/CINAHL/LILACS databases; however, after applying the exclusion criteria, only 15 articles were considered eligible for this review. The total sample was 315 participants. Among them, 77.2% were men, 13.3% were women and 9.5 uninformed. Their age ranged from 20.1 ± 2 to 47.8 ± 7.5 years. HIIT protocols included running with ergometers (n = 6), CrossFit-specific exercises (n = 2), running without ergometers (n = 3), swimming (n = 1), the Wingate test on stationary bicycles (n = 2), and cycling (n = 1). The most applied intensity controls were %vVO_2_max, “all out”, MV, MAV, Vmax, and HRreserve%. The most used markers to evaluate muscle damage were creatine kinase, myoglobin, and lactate dehydrogenase. The time for muscle damage assessment ranged from immediately post exercise to seven days. HIIT protocols were able to promote changes in markers of exercise-induced muscle damage, evidenced by increases in CK, Mb, LDH, AST, ALT, pain, and muscle circumference observed mainly immediately and 24 h after the HIIT session.

## 1. Introduction

High-intensity interval training (HIIT) is one of the training methods gaining prominence and popularity in recent years, mainly because of its efficiency and safety in individuals with different pathologies and fitness levels [1,2,3,4,5,6,7]. Generally, HIIT has significantly lower training volumes compared to moderate-intensity continuous training (MICT) [1,4,6,8,9].

In 2014, HIIT made it onto the list of fitness trends published by the American College of Sports Medicine, and since then, for the past nine years, HIIT has appeared among the top fitness trends worldwide, according to the ACSM [10]. The interest in HIIT occurs because of three main factors, commonly cited as limitations to regular physical activity, such as (1) lack of time, (2) lack of motivation, and (3) chronic illnesses that restrict work capacity while exercising [11,12].

Although without a universal definition, HIIT involves repeated sessions of short intermittent exercise, usually performed with high-intensity efforts interspersed with an active or passive recovery period [1,3,4]. HIIT can be performed using many types of equipment and exercise such as bicycles, treadmills [4,6], running [13], naval ropes [14], and even exercises that use one’s own body weight as resistance [15,16].

The HIIT prescription consists of manipulating several variables such as stimulus intensity and duration; intensity and duration of the recovery interval; exercise mode; number of repetitions; and number of sets, as well as the intensity and duration of recovery between sets. The variation in any of these parameters can affect different physiological responses caused by HIIT [3,17].

Exercise-induced muscle damage is one of the variables affected in response to intense exercise, often occurring after performing unusual exercise or a new type of exercise [18,19,20]. Symptoms of exercise-induced muscle damage usually occur within the first 24 h, peaking from 24 to 72 h, and may last from five to seven days after the exercise session [21]. Functional and blood markers are commonly used and reflect part of the different physiological processes of exercise-induced muscle damage, such as (1) loss of myofibrillar integrity (disruption and derangement of Z-discs) [22]; (2) reduction in muscle strength as a result of remodeling in the extracellular matrix or failure in excitation–contraction coupling [23,24]; (3) increased delayed-onset muscle soreness (SOR) and decreased range of motion (ROM) associated with connective tissue damage [25,26]; (4) extravasation of muscle proteins into the bloodstream such as creatine kinase (CK), lactate dehydrogenase (LDH), and myoglobin (Mb) due to damage to the cell membrane [9,27]; (5) increased limb circumference (CIR) and muscle thickness, which suggests muscle edema [28,29]; and (6) inflammatory processes that are perceived by the subject as late-onset muscle pain, weakness, limited muscle movements, and decreased performance [19,30,31]. Moreover, these conditions may affect performance in the subsequent training session.

Understanding the acute responses generated by an HIIT session, especially on exercise-induced muscle damage, can help to design strategies during its prescription regarding the HIIT modality used, the intensity and duration of the stimulus and interval, and the recovery period according to the needs during training. Although HIIT has been shown to be very effective, few studies in the literature have investigated the effect of HIIT and its settings on exercise-induced muscle damage. Thus, this study aimed to conduct a systematic review of the effect of a single HIIT session on exercise-induced muscle damage.

## 2. Materials and Methods

This systematic review sought to follow the recommendations of the PRISMA methodology and used the PROSPERO database (No. CRD42022378643). The PICOT method was used to formulate the guiding question: P—participants (individuals submitted to the HIIT session); I—intervention (pre-intervention findings); C—comparison (post-intervention findings); O—outcome (exercise-induced muscle damage parameters); and T—intervention time (single session). Thus, the research strategy sought to answer the following problem: can a single HIIT session cause exercise-induced muscle damage (EIMD)? 

The systematic search was performed using electronic databases in Medline/PubMed/Science Direct/Embase/Scielo/CINAHL/LILACS, and the following words as descriptors: “High Intensity Interval Training” OR “High-Intensity Interval Trainings” OR “Interval Training, High-Intensity” OR “Interval Trainings, High-Intensity” OR “Training, High-Intensity Interval” OR “Trainings, High-Intensity Interval” OR “High-Intensity Intermittent Exercise” OR “Exercise, High-Intensity Intermittent” OR “Exercises, High-Intensity Intermittent” OR “High-Intensity Intermittent Exercises” OR “Sprint Interval Training” OR “Sprint Interval Trainings” OR “High-Intensity Functional Training” OR “Muscle damage”, and “Exercise-induced muscle damage”. The search was conducted from 5 June to 18 September 2023. 

The following eligibility criteria were adopted: The inclusion criteria were keyword in the title and abstract; acute intervention protocol; healthy subjects; articles in English, Spanish, or Portuguese; and variables of exercise-induced muscle damage. Exclusion criteria were chronic intervention protocol, unhealthy subjects, animal studies, use of muscle recovery strategies, review articles, and case studies.

**Figure 1:**
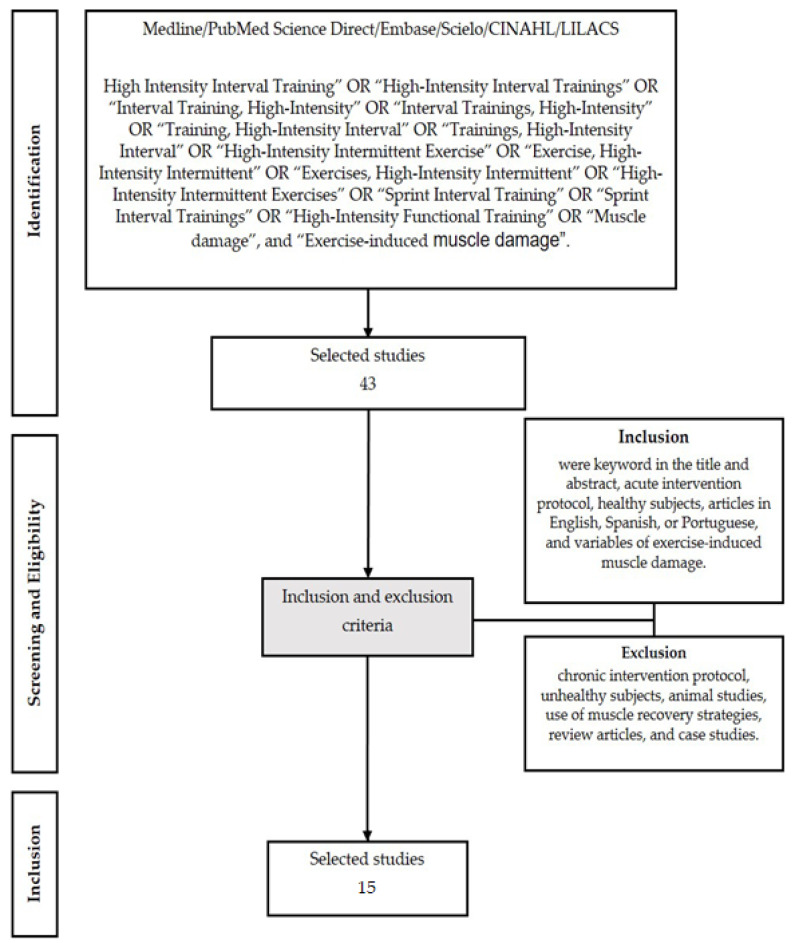
Flowchart with the stages of the methodology for selecting articles.

The selection of studies was conducted by four reviewers (C.D.F.C.L., P.V.C.Z., R.L.R., and B.M.B) by searching the databases. All search results were imported into the Rayyan software (Qatar Computing Research Institute, Qatar Foundation, Doha, Qatar) to ensure a systematic, comprehensive search and to document the selection process. One reviewer (B.M.B.) managed the Rayyan program, identifying and removing duplicate citations and ensuring an independent review of titles and abstracts (blinding the decisions of the two reviewers). C.D.F.C.L., P.V.C.Z., and R.L.R. reviewed the titles and abstracts of the shortlisted citations in the Rayyan program using a customized inclusion/exclusion checklist (chronic intervention protocol, unhealthy subjects, animal studies, use of muscle recovery strategies, review articles, and case studies). B.M. then identified discrepancies between the two reviewers using the Rayyan software and informed the reviewers of the need to establish a consensus for the selection of the studies. Full-text copies of all selected studies were obtained to acquire more details. All reviewers reviewed the full-text copies of articles to identify whether diagnostic instruments were used to identify EIMD in the subjects. Figure 1 presents the flowchart with the stages of the methodology for selecting articles. 

Three reviewers (C.D.F.C.L., P.V.C.Z., and B.M.B.) independently appraised the methodological quality of the studies using Jadad score [32] and risk of bias (RoB-2) using Cochrane tool reported in the Cochrane Collaboration handbook [33]. Differences in opinion regarding the RoB-2 and Jadad were resolved through discussion between the reviewers until reaching a consensus. If differences persisted, a third reviewer was consulted to obtain consensus through discussion or arbitrage [34]. The Jadad score consisted of three items: randomization (0–2 points), blinding (0–2 points), and dropouts and withdrawals (0–1 points). The response to each item was either “yes” (1 point) or “no” (0 points). The final score ranged from 0 to 5 points, with higher scores indicating better reporting. Studies with a Jadad score of 2 or less were considered to have low quality and those with a Jadad score of 3 or more were considered to have high quality [32]. The RoB-2 tool comprises six domains: (1) selection bias (e.g., random sequence generation and allocation concealment), (2) performance bias (e.g., blinding of participants), (3) detection bias (e.g., blinding of outcome assessment), (4) attrition bias (e.g., incomplete outcome data), (5) reporting bias (e.g., selective reporting), and (6) other biases. This tool enables researchers to assign a quality score of “high”, “low”, or “unclear” risk based on seven factors that might cause the effect of treatment to be overestimated or underestimated in individual studies. 

## 3. Results

Searches in the databases with pre-determined keywords led to the recovery of 43 records. After applying the eligibility criteria (Figure 1), 15 articles were included in this SR.

We found one study published in 2010, six studies from 2015 to 2017, one in 2018, three in 2019, one in 2020, one in 2021, and two in 2023. Table 1 shows the characteristics of the selected studies. All of them were published in English. The journal impact factor varied from 1.150, the lowest value, to 5.200, the highest value. 

**Figure 2:**
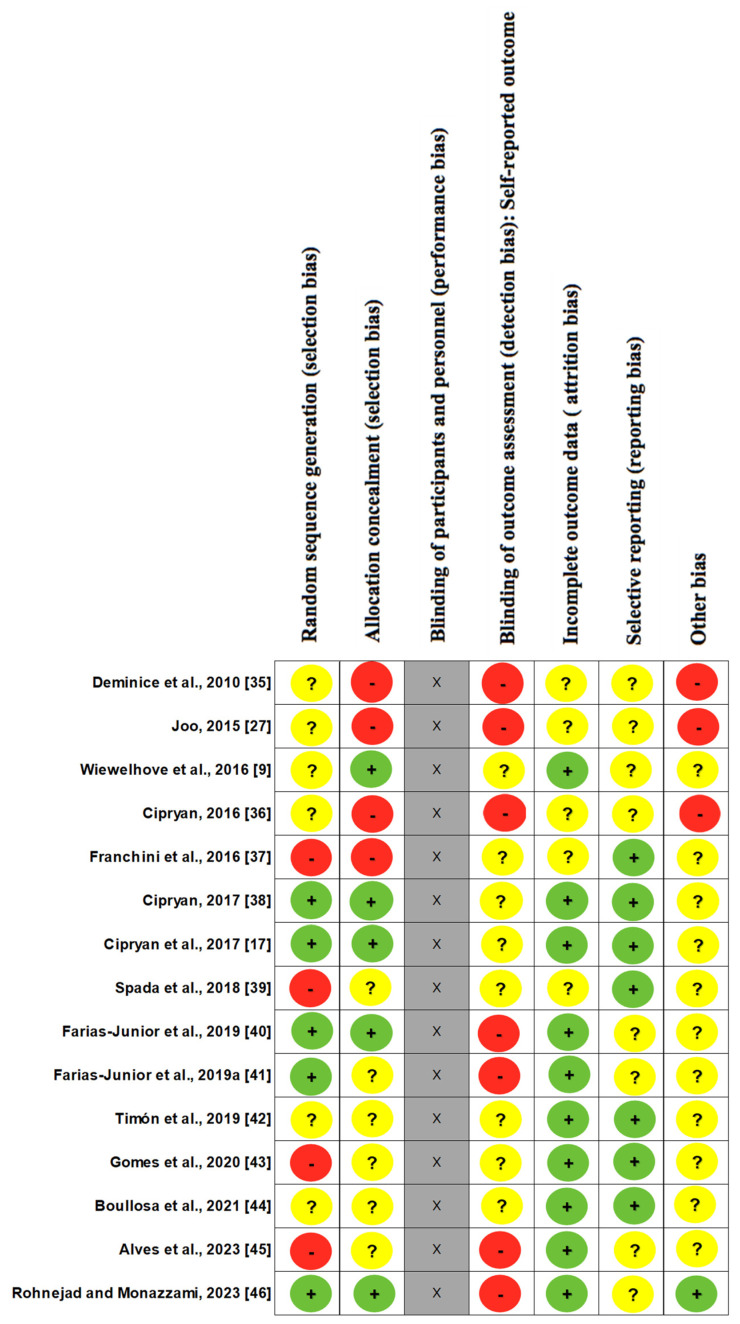
Cochrane risk of bias for individual studies (Roob-2 evaluation). Included studies falling under low risk (green), unclear risk (yellow), and high risk (red) are shown for each of the seven. Item blinding of participants and personnel (performance bias) was not evaluated (gray).

Table 2 and Figure 2 show the evaluation of methodological quality and bias risk. The average score on the Jada quality scale (Table 2) was 3.53 ± 0.74 points, with nine studies with 3 points, four with 4 points, and two with 5 points showing high quality. 

Regarding the Roob-2 assessment (Figure 2), of the 15 studies referring to random sequence generation, 5 studies showed low risk, 6 studies left it unclear, and 4 studies showed high risk. In relation to blinding and allocation, 5 studies showed low risk, 6 studies left it unclear, and 4 studies showed high risk. Regarding the blinding of participants, the evaluation was not carried out, due to the studies being about exercises, and there was no possibility of blinding. No study was evaluated as low risk for blind evaluation of the results, 7 showed high risk, and 8 left it unclear; however, regarding the acceptance of incomplete results, 10 studies showed low risk and 5 studies left it unclear. In relation to selective reports, 7 showed low risk and 8 left it unclear. Finally, in relation to other biases, 1 study showed low risk, 11 left it unclear, and 3 showed high risk. No discrepancies were found in the analyses provided by the researchers.

As Table 3 shows, the selected studies totaled 315 participants, two with sedentary individuals. Among them, 77.2% were men, 13.3% were women and 9.5% uninformed. Their mean age ranged from 20.1 ± 2 to 47.8 ± 7.5 years. The HIIT protocols used were running using ergometers (6), and CrossFit common exercises such as burpees, toes to bar, wall ball, power clean, fixed bar, air bend and squat (3), swimming (1), running without ergometers (2), the Wingate test on stationary bicycles (2), and cycling (1). The intensity controls were %vVO_2_max, “all out”, MV, MAV, Vmax, and HRreserve%.

Blood markers and subjective and functional parameters were used to evaluate exercise-induced muscle damage. The blood markers used were creatine kinase (CK), myoglobin (Mb), lactate dehydrogenase (LDH), aspartate aminotransferase (AST), and alanine aminotransferase (ALT). Thigh circumference was used as an objective marker; perception of muscle pain and sensitivity was used as subjective markers; and the functional markers used were maximum voluntary contraction (MVC), countermovement jump (CMJ), countermovement vertical jump height (CVJH), plank test (PT), pressure–pain threshold (PPT), pressure–pain tolerance (PPTol), and EVA—perceived pain intensity (PPI). From our selected studies, 13 used CK and 5 used Mb and LDH as blood markers for muscle damage. Pain perception was used in seven studies. Other, less used parameters were pressure–pain threshold, four studies; countermovement jump, AST, and ALT, three studies; pressure–pain tolerance, four studies; and maximum voluntary contraction, muscle circumference, and plank test, three studies. The analysis times were immediately after protocols (POS), and 30 min; 1, 2, 3, 4, 24, 48, and 72 h; and seven days after exercise. POS and 24 h were the most used times for evaluation. 

According to the selected studies, the diverse variations in HIIT protocols were able to promote changes in muscle damage markers after exercise in the analyzed subjects. Table 4 shows the studies’ summary details.

## 4. Discussion

This study aimed to describe, by means of a systematic review, the effects of a single HIIT session on markers of exercise-induced muscle damage. Of the 15 studies analyzed, none was evaluated with low methodological quality. Although we found 43 studies, only 15 of them evaluated the effect of an HIIT session on muscle damage markers. Some factors may directly influence exercise-induced muscle damage, such as type of contraction, degree of training, and intensity of exercise [18,20,47]. Muscle damage usually occurs with the practice of strenuous or unusual exercises. The type of contraction is one of the factors that may influence muscle damage, in which eccentric actions caused a greater response to muscle damage when compared to concentric actions [20].

The degree of training should also be considered; thus, the literature shows that trained subjects present smaller changes in muscle function, circumference of the limbs, and activity of enzymes commonly used to assess muscle damage when compared with untrained subjects [47]. Except one, all our selected studies dealt with most trained subjects, and some of them were used to high-intensity exercise in their training routine. These studies showed that even with a greater number of trained volunteers, muscle damage still occurred [35].

The exercise intensity may also affect the magnitude of exercise-induced muscle damage [18]. Despite the subjects’ degree of training, the high-intensity HIIT protocols may compromise muscle fiber, leading to injuries [17,18]. Furthermore, we can hypothesize that the factor intensity of exercise overlaps the degree of training. Gomes et al. [43] evaluated an HIIT session in beginners and experienced CrossFit subjects. Regardless of their conditioning level, CK levels increased immediately after the session and remained elevated for up to 24 h, with no distinction between the groups. Cipryan [36] evaluated the effect of HIIT on individuals in different degrees of training. Although the HIIT protocol increased muscle damage markers, increases in CK and Mb were higher in moderately trained and untrained subjects compared to well-trained subjects.

In the study by Deminice et al. [35], all subjects were athletes and had been training regularly for more than five years, six days per week, about 2.5 h per day, as well as participating in national competitions and being familiar with the HIIT series in their training routine. After the HIIT session with maximum effort intensity, CK increased [28].

Joo [27] evaluated moderately trained individuals used to often performing high-intensity exercises. They evaluated CK, Mb, PMP, and MVC values post exercise and over seven days. Mb and PMP increased 24 and 48 h after exercise, returning to baseline values at 72 h. Despite the results, the authors suggest that the protocol caused acute fatigue effects. However, studies show that after exercise-induced muscle damage, Mb immediately increased [17,36,38], as well as muscle pain after 24 and 48 h [18,19,48].

During HIIT, eccentric actions may cause exercise-induced muscle damage [27,49,50]. Muscle damage can be caused by either or both metabolic or mechanical stress, depending on the mode, intensity, and duration of exercise, as well as the individuals’ training status [49]. Wiewelhove et al. [9] show that when the structure and characteristics of HIIT protocols are changed, even with a similar duration, distinct changes in muscle damage markers can occur. Additionally, they affirm that sprint protocols induce greater damage and muscle pain compared to interval protocols of longer duration and sub-maximum intensity. These results shall be considered when planning and recovering from high-intensity interval protocols.

Franchini et al. [37] observed an increase in CK, LDH, AST, and ALT concentrations after the Wingate test in a stationary bicycle [37]. Cipryan et al. [17] showed increased Mb after HIIT sessions with short and long intervals without distinguishing between them. Furthermore, such results failed to show any differences between athletes trained in endurance and sprint. Cipryan [36], to extend the results, evaluated the effects of three different HIIT protocols in moderately trained subjects, with identical external work on muscle damage markers. The results showed that all three protocols were able to immediately increase the levels of muscle damage markers (CK, Mb, and LDH) in the blood. 

In the study by Spada et al. [39] with trained volunteers and after an acute session of an HIIT protocol in a study by Tabata et al. [51], muscle injury occurred due to the significant increase in CK immediately after exercise, which was three times higher after 24 h, and in Mb two hours after exercise, which maintained its high value 24 h after the session.

Boullosa et al. [44], in their study of physically active men after an acute HIIT session involving eccentric and concentric cycling protocols, also observed a significant increase in CK immediately after the two protocols and its normalization 24 h later. However, they reported differences between cycling protocols when evaluating other markers of muscle damage, such as the visual analog scale (VAS) and thigh circumference, in which VAS and thigh circumference changed only 24 h after the eccentric cycling protocol.

Moreover, two studies evaluated an HIIT session with exercises commonly used in a CrossFit session. In both studies, CK increased immediately and 24 h after the HIIT session [42,43]. In a study by Timón et al. [42], the subjects had experience in CrossFit for at least one year of training two days per week. In addition, the usual practice of high-intensity CrossFit sessions could cause the high levels of LDH and CK (above normal reference values). In summary, the high-intensity characteristic of HIIT protocols induced a certain degree of muscle damage in trained subjects.

In contrast, it is important to mention that some studies do not indicate muscle damage [40,41,45,46,52]. Rohnejad and Monazzam [46] demonstrated an increase in the levels of muscle damage markers 1 h, 24 h, and 48 h after HIIT in middle-aged men; however, the authors concluded that the practice was not severe, as recovery periods of muscle damage markers are faster. Farias-Júnior et al. [41] observed that in overweight participants, a modest increase in muscle damage after HIIT was insufficient to change performance and capacity. Alves et al. [45] showed no changes in muscle damage markers after 24 and 48 h in recreationally trained men after LV-HIIT sessions with different work–recovery durations. Farias-júnior et al. [40] observed that HIIT when compared with continuous exercise promoted similar muscle damage 24 h after exercise, but this did not cause movement restriction when performing daily activities.

Functional parameters are used by several authors as an indirect method to evaluate EIMD [29,52,53,54,55]. There are many options commonly incorporated into EIMD studies that can be accessible to coaches and fitness instructors [22,26,29,52,53,54,55]. Since HIIT protocols were able to promote changes in markers of exercise-induced muscle damage, monitoring post-session responses can be accessible and help coaches and fitness instructors improve design strategies for HIIT prescription. In addition, some considerations should be mentioned. First, curiously, although some studies had used other methods such as ultrasound [29,52], muscular biopsy [22,53,54], and molecular parameters [22,53,54], no studies found in our search analyzed the EIMD using these methods after HIIT. It could be a new methodological strategy to improve knowledge about EIMD and HIIT.

## 5. Conclusions

HIIT protocols were able to promote changes in markers of exercise-induced muscle damage, evidenced by increases in CK, Mb, LDH, AST, ALT, pain, and muscle circumference observed mainly immediately and 24 h after the HIIT session. CK and Mb were the most frequently used markers regardless of the protocols used in the exercise sessions. Factors such as high intensity during exercises and the metabolic stress generated during HIIT may contribute to the occurrence of damage. Prescribing HIIT is a complex process and must be managed appropriately according to each goal. Additionally, several variables, such as the type of exercise used, can be employed, affecting the post-exercise response and its adaptations.

## Figures and Tables

**Table 1:** General characteristics of the selected studies.

Authors	Language	Journal	IF
Deminice et al., 2010 [35]	English	*The Journal of Sports Medicine and Physical Fitness*	1.432
Joo, 2015 [27]	English	*Journal of Exercise Rehabilitation*	1.170
Wiewelhove et al., 2016 [9]	English	*The Journal of Sports Medicine and Physical Fitness*	1.432
Cipryan, 2016 [36]	English	*Journal of Sport and Health Science*	5.200
Franchini et al., 2016 [37]	English	*Frontiers in Physiology*	3.367
Cyprian, 2017 [38]	English	*Journal of Human Kinetics*	1.664
Cipryan et al., 2017 [17]	English	*Journal of Sports Science and Medicine*	1.806
Spada et al., 2018 [39]	English	*Plos One*	2.740
Farias-Junior et al., 2019 [40]	English	*The Journal of Strength and Conditioning Research*	3.200
Farias-Junior et al., 2019a [41]	English	*Physiology & Behavior*	3.742
Timón et al., 2019 [42]	English	*Biology of Sport*	2.000
Gomes et al., 2020 [43]	English	*Plos One*	2.740
Boullosa et al., 2021 [44]	English	*International Journal of Environmental Research and Public Health*	4.614
Alves et al., 2023 [45]	English	*Research Quarterly for Exercise and Sport*	2.098
Rohnejad and Monazzami, 2023 [46]	English	*Apunts Sports Medicine*	1.150

**Table 2:** The methodological quality of the studies according to Jadad scale.

Study	Was the Study Described as Randomized?	Was There a Description of Randomization? Was it Adequate?	Were There Comparisons and Results?	Was There a Description of Comparisons and Results? Were They Adequate?	Was There a Description of Withdrawals and Dropouts?	Total
Deminice et al., 2010 [35]	0	0	1	1	1	3
Joo, 2015 [27]	0	0	1	1	1	3
Wiewelhove et al., 2016 [9]	0	1	1	1	1	4
Cipryan, 2016 [36]	0	0	1	1	1	3
Franchini et al., 2016 [37]	0	1	1	1	1	4
Cipryan, 2017 [38]	0	0	1	1	1	3
Cipryan et al., 2017 [17]	0	0	1	1	1	3
Spada et al., 2018 [39]	0	0	1	1	1	3
Farias-Junior et al., 2019 [40]	1	1	1	1	1	5
Farias-Junior et al., 2019a [41]	1	0	1	1	1	4
Timón et al., 2019 [42]	0	1	1	1	1	4
Gomes et al., 2020 [43]	0	0	1	1	1	3
Boullosa et al., 2021 [44]	0	0	1	1	1	3
Alves et al., 2023 [45]	0	0	1	1	1	3
Rohnejad and Monazzami, 2023 [46]	1	1	1	1	1	5

**Table 3:** General characteristics of the studies.

Reference	Subjects/Sample	Age (Years)	VO_2_	HIIT Protocol	Intensity
Deminice et al., 2010 [35]	A total of 10 well-trained swimmers among the top 10 best Brazilian swimmers in their styles (men n = 8; women n = 2), familiar with HIIT series in their training routine	20 ± 2	N/A	A set of 8 maximal swims over 100 m per their style specialty, with 10 min rest.	Maximum effort
Joo, 2015 [27]	A total of 10 healthy, moderately trained men used to frequent high-intensity exercise	31 ± 7.1	VO_2_max 58 ± 7.1 mL kg min	A total of 8 sets of 3 min jogging sessions on a treadmill, interspersed with 3 min active intervals (1.5 min at 25% VO_2_max and 1.5 min at 50% VO_2_max).	90% VO_2_max
Wiewelhove et al., 2016 [9]	A total of 16 well-trained male athletes from intermittent sports (tennis, handball, and soccer)	24.6 ± 2.7	VO_2_max 58.3 ± 5.9 mL kg min	A total of five different HIIT protocols, separated by six days each, were performed. HIIT-P240: 4 sets of 4 min with 3 min passive interval (2/1 work/rest ratio). HIIT-P120: 7 sets of 2 min with 2 min passive interval (1/1 work/rest ratio), 40 m come-and-go run test. HIIT-P30: 2 blocks with 10 sets of 30 s with 45 s interval and 3 min passive recovery between the blocks (1/2 work/rest ratio). HIIT-P15: 3 blocks with 9 sets of 15 s with 30 s interval and 3 min of passive recovery between blocks (1/4 work/rest ratio), sprint straight. HIIT-P5: 4 blocks of 6 sets of 5 s with 25 s interval and 5 min of passive recovery between blocks (1/12 work/rest ratio).	%V 30–15 intermittent fitness test HIIT-P240—80% HIIT-P120—85% HIIT-P30—90% HIIT-P15—95% HIIT-P5—all out
Cipryan, 2016 [36]	The sample consisted of 30 healthy young subjects distributed in well trained (WT; n = 11; h/week 12.00 ± 5.89), moderately trained (MT; n = 10; h/week 6.05 ± 2.22), untrained (UT; n = 9; no intentional sports activities)	WT: 24.18 ± 1.80 MT: 24.18 ± 1.80 UT: 24.44 ± 2.54	VO_2_ max WT: 61.39 ± 3.63 mL kg min MT: 53.46 ± 2.80 mL kg min UT: 47.21 ± 3.98 mL kg min	All participants performed a 30 min HIIT composed of 6 × 2 min interval exercise with work-to-rest ratio = 1.	100% vVO_2_max
Franchini et al., 2016 [37]	The sample consisted of 35 male judo athletes divided into HIIT in stationary bicycle for lower limbs (HIIT-L; n = 9), HIIT in stationary bicycle for upper limbs (HIIT-U; n = 9), Uchi-Komi judo technique (HIIT-UK; n = 9), and control (C; n = 8)	HIIT-L: 22.3 ± 5.2 HIIT-U: 23.6 ± 6.7 HIIT-UK: 23.4 ± 4.2 control: 26.4 ± 7.0	VO_2_ peak Gradual maximal upper limb stationary bicycle test for each group (PRE values) HIIT-L: 2.78 ± 0.41 L.min HIIT-U: 3.10 ± 0.70 L.min HIIT-UK: 3.16 ± 0.30 L.min control: 2.86 ± 0.37 L.min Gradual maximal lower limb stationary bicycle test for each group (PRE values) HIIT-L: 3.62 ± 0.50 L.min HIIT-U: 3.82 ± 0.59 L.min HIIT-UK: 3.87 ± 0.44 L.min control: 3.56 ± 0.49 L.min	Tests on the stationary bicycle with 70 rpm fixed cadence for lower limbs and 90 rpm for upper limbs, totaling 22 min/session. Session divided into 2 HIIT blocks, each block lasting 4 min (10 times/20 s effort and 10 s break), and 5 min rest between each block.	All out
Cyprian, 2017 [38]	A total of 12 moderately trained men participated in three HIIT trials	22.8 ± 1.7	VO_2_max 57.2 ± 6.3 mL kg min	The three different HIIT protocols were performed on a treadmill with work/rest ratio = 1 (HIIT 15 s/15 s, HIIT 30 s/30 s, and HIIT 60 s/60 s), the total duration was 12 min with identical external work with active recovery of 6 min at 60% vVO_2_max.	100% vVO_2_max
Cipryan et al., 2017 [17]	In total, 16 highly trained men were divided into endurance athletes (E = n = 8; h/week 13.9 ± 4.0) and sprint athletes (S = n = 8; h/week 9.9 ± 1.9), both groups performed 3 HIIT protocols	E: 22.1 ± 2.5 S: 22.9 ± 3.5	VO_2_max E: 66.2 ± 5.0 mL kg min S: 56.8 ± 5.0 mL kg min	A total of two HIIT protocols were performed on a treadmill. The 3 min HIIT consisted of 4 sets of 3 min of work with 3 min of passive recovery interval. The 30 s HIIT consisted of 21 sets of 30 s of work with 30 s of passive recovery interval. The control group ran for 21 min.	3 min HIIT: 100% vVO_2_max 30 s HIIT: 100% vVO_2_max C: 50% vVO_2_max
Spada et al., 2018 [39]	A total of 58 healthy volunteers (29 men and 29 women), able to correctly perform the prescribed exercise, and with serum and urinary laboratory parameters within normal ranges, performed a high-intensity interval resistance training (HIIRT) session.	24 (21–28)	N/A	The HIIRT session consisted of 8 sets of squats with the fastest speed and the highest number of repetitions achievable for 20 s with 10 s of rest between sets.	Maximum effort
Farias-júnior et al., 2019 [40]	The sample consisted of 15 untrained healthy males	25.1 ± 4.4	N/A	The low-volume HIIE consisted of 10 × 60 s work bouts interspersed with 60 s of active recovery at 30% of MV.	90% of Maximal velocity (MV)
Farias-júnior et al., 2019a [41]	The sample consisted of 20 overweight inactive men	28.9 ± 5.0	VO_2_pico 39.0 ± 4.1	The HIIE consisted of 10 × 1 min intervals interspersed with 1 min of passive recovery.	100% of Vmax
Timón et al., 2019 [42]	A total of 12 trained men and CrossFit practitioners completed two modalities of WODs on separate days: WOD1 (as many rounds as possible) and WOD2 (rounds for time)	30.4 ± 5.37	VO_2_max 47.8 ± 3.63 mL kg min	They practiced two modalities of workout of the day (WODs) on separate days. WOD1: as many rounds as possible of burpees and toes to bar with increasing repetitions (1-1, 2-2, 3-3,...) in five minutes. WOD2: 3 blocks of 20 wall ball (9 kg) repetitions and then 20 power clean repetitions (load of 40% of 1 RM) in the shortest time possible.	N.I
Gomes et al., 2020 [43]	A total of 23 subjects, 12 men and 11 women, were divided into experienced (EXP: ≥18 months of experience; n = 13) and beginners (BEG: 3–8 months experience; n = 10) and were submitted to a specific protocol of the modality	EXP: 31.1 ± 4.9 BEG: 30.9 ± 4.8 ALL: 31.0 ± 4.8	VO_2_max EXP: 40.7 ± 1.8 mL kg min BEG: 39.2 ± 1.4 mL kg min ALL: 40.0 ± 1.7 mL kg min	The high-intensity functional training session (HIFT) WOD developed was called “Cindy”. This WOD consisted of as many rounds as possible of 5 pull-ups, 10 push-ups, and 15 air squats in 20 min.	All out
Boullosa et al., 2021 [44]	The sample consisted of 12 physically active men involved in recreational endurance sports	23.4 ± 2.8	VO_2_max: ≥90% of the maximum predicted heart rate for age (HRmax)	A total of 8 maximal efforts for 5 s, with 55 s of active recovery interval at 80 rpm, in concentric vs. eccentric cycling.	All out
Alves et al., 2023 [45]	The sample consisted of 24 trained adult males	22.3 ± 2.9	N.I	Two LV-HIIT sessions: The 60/60 s LV-HIIT protocol consisted of 10 × 60 s of maximal aerobic speed on treadmill interspersed by 60 s of passive recovery. The 30/30 s LV-HIIT protocol with 20 × 30 s of maximal aerobic speed on treadmill interspersed by 30 s of passive recovery.	100% Vmax
Rohnejad and Monazzami, 2023 [46]	The sample consisted of 22 overweight middle-aged active men	Control: 47.80 ± 7.50 HIIT group: 45.90 ± 6.17	VO_2_max Control: 28.5 1± 1.55 mL kg min HIIT group: 28.14 ± 1.30 mL kg min	The HIIT training program consisted of intermittent running for 30 s, 30 s of active recovery at 50% aerobic speed (4 sets, 4 rounds, and 5 min of passive recovery between each round).	100% Maximal aerobic velocity (MAV)

**Table 4:** Characteristics and timing of analysis of muscle damage markers.

Reference	Damage Markers	POS	30 min	1 h	2 h	3 h	4 h	24 h	48 h	72 h	7 Days	Conclusion
Deminice et al., 2010 [35]	1. CK	↑ CK	N/A	N/A	N/A	N/A	N/A	N/A	N/A	N/A	N/A	Proposed session-specific HIIT induces increased creatine kinase in competitive swimmers.
Joo, 2015 [27]	1. CK * 2. Mb 3. Pain-VAS 4. Muscle Pain Sensitivity (distal myotendinous junction and middle belly of rectus femoris) * 5. MVC *	↑ Mb	N/A	N/A	N/A	N/A	N/A	↑ Mb	↔ Mb ↑ Pain-VAS	↔ Mb ↔ Pain-VAS	↔ Mb ↑ Pain-VAS	The results show that, in moderately trained subjects used to high-intensity exercise, the exercise protocol used in this study was able to increase post-exercise myoglobin levels as well as muscle pain perception 48 h after the protocol. No other marker changed.
Wiewelhove et al., 2016 [9]	1. CK 2. Pain-VAS * 3. CMJ	N/A	HIIT-P240 * CK HIIT-5 ↓ CMJ	N/A	N/A	N/A	N/A	HIIT-P240 ↑ CK HIIT-5 ↑ CK ↓ CMJ	N/A	N/A	N/A	The HIIT-P240 straight running and the HIIT-P5 sprint showed an increase in CK 24 h after exercise. HIIT-P5 showed a CMJ reduction 30 min and 24 h after exercise, which suggests that short intervals of high-intensity training possibly cause greater muscle damage compared to long intervals of submaximal-intensity training.
Cipryan, 2016 [36]	1. CK 2. Mb	WT ↑ CK ↑ Mb MT ↑ CK ↑ Mb UT ↑ CK ↑ Mb	N/A	N/A	WT ↔ CK ↑ Mb MT ↑ CK ↑ Mb UT ↑ CK ↑ Mb	N/A	WT ↔ CK ↑ Mb MT ↑ CK ↑ Mb UT ↑ CK ↑ Mb	N/A	N/A	N/A	N/A	Although the HIIT protocol increased markers of exercise-induced muscle damage, CK and Mb increases were less pronounced in well-trained athletes compared to moderately trained or untrained individuals.
Fanchini et al., 2016 [37]	1. CK 2. LDH 3. AST 4. ALT	Wingate test values in the stationary bicycle performed before the upper and lower limbs training period. HIIT-L ↑ CK ↑ LDH ↑ AST ↑ ALT All vs. PRE for both tests HIIT-U ↑ CK ↑ LDH ↑ AST ↑ ALT All vs. PRE for both tests HIIT-UK ↑ CK ↑ LDH ↑ AST ↑ ALT All vs. PRE for both tests	N/A	N/A	N/A	N/A	N/A	N/A	N/A	N/A	N/A	Both Wingate tests in stationary bicycle (lower and upper segment) increased muscle damage markers (CK, LDH, AST, and ALT) compared to pre-test in HIIT-I, HIIT-S, and HIIT-UK groups.
Cyprian, 2017 [38]	1. CK 2. Mb 3. LDH	HIIT 15 s/15 s ↑ CK ↑ Mb ↑ LDH HIIT 30 s/30 s ↑ CK ↑ Mb ↑ LDH HIIT 60 s/60 s ↑ CK ↑ Mb ↑ LDH	N/A	N/A	N/A	HIIT 15 s/15 s ↑ CK ↑ Mb ↑ LDH HIIT 30 s/30 s ↔ CK ↑ Mb ↑ LDH HIIT 60 s/60 s ↔ CK ↑ Mb ↑ LDH	N/A	HIIT 15 s/15 s ↑ CK ↔ Mb ↑ LDH HIIT 30 s/30 s ↑ CK ↔ Mb ↑ LDH HIIT 60 s/60 s ↑ CK ↑ Mb ↑ LDH	N/A	N/A	N/A	All three HIIT protocols with short intervals and fixed external work caused an immediate elevation in muscle damage markers in circulation. However, these changes differed, prejudicing to assess the magnitude of exercise-induced muscle damage. The HIIT 30 s/30 s protocol showed a lower response in Mb.
Cipryan et al., 2017 [17]	1. CK * 2. Mb	For both ET and ST athletes: 3 min HIIT ↑ Mb HIIT-30 s ↑ Mb	N/A	For both ET and ST athletes: 3 min HIIT ↑ Mb HIIT-30 s ↑ Mb	N/A	For both ET and ST athletes: 3 min HIIT ↑ Mb HIIT-30 s ↑ Mb	N/A	N/A	N/A	N/A	N/A	Markers of muscle damage monitored during the initial recovery failed to show any differences between individuals trained in endurance and sprint. Despite this, Mb values showed a moderate response 1 h and 3 h after the 30 min and 30 s HIIT session. The control group showed no change in markers.
Spada et al., 2018 [39]	1. CK 2. Mb 3. Pain-Borg CR10	N/A	N/A	N/A	↑ CK ↑ Mb ↑ Pain	N/A	N/A	↑ CK ↑ Mb ↑ Pain	N/A	N/A	N/A	A single session of HIIT in healthy and young individuals caused increases in CK, Mb, and pain, indicating the occurrence of muscle damage.
Farias-Junior et al., 2019 [40]	1. PPT 2. PPTol 3. PPI Muscles analyzed: rectus femoris, biceps femoris, and gastrocnemius	N/A	N/A	N/A	N/A	N/A	N/A	HIIE RF ↑ PPI BF ↑ PPI G ↓ PPTol	N/A	N/A	N/A	Low-volume HIIE session elicited mild DOMS 24 h post exercise in untrained healthy males, which was similar to the traditional CE session.
Farias-Junior et al., 2019a [41]	1. CK 2. LDH 3. PPT 4. PPTol 5. EVA-PPI Muscles analyzed: rectus femoris, biceps femoris, and gastrocnemius	N/A	N/A	N/A	N/A	N/A	N/A	↑ CK G ↓ PPTol	↑ CK BF ↑ PPI G ↔ PPTol	N/A	N/A	The subjects showed modest exercise-induced muscle damage for all individuals.
Timón et al., 2019 [42]	1. CK 2. LDH * 3. AST 4. ALT 5. CMJ * 6. PT	WOD1 ↑ CK ↑ AST ↑ ALT ↓ TP WOD2 ↑ CK ↑ AST ↑ ALT ↓ TP	N/A	N/A	N/A	N/A	N/A	WOD1 ↑ CK ↔ AST ↔ ALT ↓ PT WOD2 ↑ CK ↑ AST ↑ ALT ↓ TP	WOD1 ↔ CK ↔ AST ↔ ALT ↔ PT WOD2 ↔ CK ↔ AST ↔ ALT ↔ PT	N/A	N/A	The effort intensity during WOD2 was higher than during WOD1. The performance of both CrossFit sessions (WOD1 and 2) caused significant changes in transaminases, markers of muscle damage, and reduction in physical performance. All values returned to baseline values in 48 h.
Gomes et al., 2020 [43]	1. CK	EXP ↑ CK BEG ↑ CK ALL ↑ CK	EXP ↑ CK BEG ↑ CK ALL ↑ CK	N/A	N/A	N/A	N/A	EXP ↑ CK BEG ↑ CK ALL ↑ CK	N/A	N/A	N/A	A single HIFT session significantly increased CK levels in both EXPs and BEGs.
Boullosa et al., 2021 [44]	1. CK 2. Pain–VAS 3. MC	Concentric protocol ↑ CK Eccentric protocol * CK	N/A	N/A	N/A	N/A	N/A	Concentric protocol ↔ CK * Pain–VAS * TC Eccentric protocol ↑ CK ↑ Pain–VAS ↑ MC	N/A	N/A	N/A	Single-session HIIT protocols are able to change damage markers mainly within 24 h.
Alves et al., 2023 [45]	1. Countermovement vertical jump height (CVJH) * 2. PPT * 3. PPTol * 4. EVA–PPI * Muscles analyzed: rectus femoris (RF), biceps femoris (BF), and gastrocnemius (G).	N/A	N/A	N/A	N/A	N/A	N/A	For both groups (60/60 LV-HIIT and 30/30 LV-HIIT) No change	For both groups (60/60 LV-HIIT and 30/30 LV-HIIT) No change	N/A	N/A	The LV-HIIT sessions with different work–recovery durations (i.e., 10 × 60 s or 20 × 30 s at 100% of Vmax), matched by work–recovery ratio and total work performed (i.e., 1:1 and 10 min, respectively), elicit nonsignificant changes in exercise-induced muscle damage markers (i.e., DOMS and CVJH) following 24 and 48 h in recreationally trained men.
Rohnejad and Monazzami, 2023 [46]	1. CK 2. LDH 3. AST 4. ALT	N/A	N/A	↑ CK ↑ LDH ↑ ALT ↑ AST	N/A	N/A	N/A	↑ CK ↔ LDH ↔ ALT ↔ AST	↑ CK ↔ LDH ↔ ALT ↔ AST	N/A	N/A	The findings revealed that HIIT training led to a significant change in muscle damage variables in the training group in one hour after the training compared to the pre-test. Furthermore, the results showed that at 24 h and 48 h after training, no difference was observed between the training and control groups in the variables of LDH, ALT, and AST.

↑, Increase; ↓, Decrease; ↔, Return to baseline values; *, there was no change in the evaluated variable; N/A, not evaluated at this time; CK, creatine kinase; Mb, myoglobin; Pain-VAS, perception of muscle pain—visual analogic scale; MVC, maximum voluntary contraction; CMJ, countermovement jump; CVJH, countermovement vertical jump height; LDH, lactate dehydrogenase; AST, aspartate aminotransferase; ALT, alanine aminotransferase; MC, muscle circumference; PT, plank test; WT, well trained; MT, moderately trained; UT, untrained; ET, endurance trained; ST, sprint trained; BEG, beginner; EXP, experienced; PPT, pressure–pain threshold (minimal pressure that induced pain); PPTol, pressure–pain tolerance (maximal pressure supported by the participant, i.e., highest level of pain tolerated by the participant); PPI, EVA—perceived pain intensity (was assessed using a visual analog scale, with “no pain” at one end of a 100 mm line and “worst possible pain” at the other).

## Data Availability

Data are contained within the article.
